# Influence of Neuroticism on Depressive Symptoms Among Chinese Adolescents: The Mediation Effects of Cognitive Emotion Regulation Strategies

**DOI:** 10.3389/fpsyt.2020.00420

**Published:** 2020-05-14

**Authors:** Chengwei Liu, Liang Chen, Sanmei Chen

**Affiliations:** ^1^School of Education, Hunan University of Science and Technology, Xiangtan, China; ^2^School of Marxism, University of Science and Technology Liaoning, Anshan, China

**Keywords:** neuroticism, depressive symptoms, cognitive emotion regulation, multigroup mediating effect, adolescents

## Abstract

**Objective:**

This study aims to explore the multilevel mediation effects of cognitive emotion regulation strategies (CERS) on the link between neuroticism and depressive symptoms among Chinese adolescents.

**Method:**

A total of 1,265 Chinese adolescents were surveyed using the Chinese version of the Cognitive Emotion Regulation Questionnaire (CERQ-C), the neuroticism scale of the Chinese children’s version of the Eysenck Personality Questionnaire (EPQ-Ck), and the Chinese Children’s Depressive symptoms Inventory (CDI-C). Partial correlation analyses, multigroup confirmatory factor analyses, and structural equation modeling were used.

**Results:**

(1) Neuroticism had significant, positive correlations with maladaptive CERS strategies (self-blame, acceptance, rumination, catastrophizing, and other-blame) and depressive symptoms (*ps* < 0.001). Adaptive CERS strategies (positive refocusing, refocus on planning, positive reappraisal, and putting into perspective) had significant, negative correlations with neuroticism and depressive symptoms (*ps* < 0.001). (2) Neuroticism and CERS strategies significantly predicted depressive symptoms. CERS strategies played partially mediating roles in the relationship between adolescents’ neuroticism and depressive symptoms.

**Conclusion:**

CERS strategies have partial multilevel mediation effects on the link between neuroticism and depressive symptoms.

## Introduction

Gross (1) described emotion as the pattern of synergistic reactions produced by individuals in the face of important opportunities and challenges in life and noted that emotion can manifest as physiological responses, subjective experiences, and expressions of behavior (1). As a relatively stable long-term personality trait, neuroticism is most closely related to negative emotions. People with higher levels of neuroticism commonly have stress reactions with negative emotions while experiencing the lasting effects of these negative emotions. Several studies have confirmed that neuroticism is closely related to depressive symptoms and anxiety. For example, Hankin et al. contended that neuroticism could effectively predict depressive symptoms in adults and minors. Neuroticism scores were directly proportional to the severity of individual depressive symptoms (2). Similar findings were obtained by Steca et al., who affirmed that the severity of depressive symptoms was correlated with the individual’s neurotic behavioral scores in clinical studies of adult depressive symptoms (3). Neuroticism also plays an important role in the development of depression. In particular, scholars have found that neuroticism not only is significantly related to depressive symptoms but also has significant predictive effects on depressive symptoms (4). In short, existing studies have clearly suggested that neuroticism is a risk factor for experiencing depressive symptoms.

Numerous studies have shown that emotion regulation mediates the relationship between neuroticism and depressive symptoms (5). The systematic use of maladaptive CERS and the development of emotional disorders can exert significant impacts on the initiation and exacerbation of depressive symptoms (6). As an individual ages, his/her emotional regulation becomes more complicated and transitions from a behavior-oriented emotional regulation strategy to a cognitive-based emotional regulation strategy. Adolescence is a critical period of development of the cognitive emotion regulation strategy (7). Different CERS might have different effects in down-regulating negative moods. That is, even if a person has a clear bias toward experiencing a negative mood (i.e., high neuroticism), this person might not suffer from a sustained negative affect if he/she has good ability of emotion regulation. Neuroticism seems to correlate with the severity of depressive symptoms because of its correlation with greater use, with persistence and rigidity, of maladaptive emotion regulation strategies (8, 9). That is, the use of different CERS might mediate the effect of neuroticism on depressive symptoms. Studies have found that rumination partially mediates the relationship of college students’ neuroticism with anxiety and depressive symptoms (10). This mediating effect was also confirmed for high school students in China (11, 12). Other studies have found that neuroticism is negatively related to adaptive CERS and that a cognitive reappraisal strategy has a moderating effect on the relationship between neuroticism and depressive symptoms (13). In addition, a study with adults found that cognitive and behavioral emotional regulation strategies directly affect the relationship between neuroticism and depressive symptoms (5). Through cognitive emotional regulation training, such as mindfulness, the relationship between neuroticism and depressive symptoms can be effectively regulated (14).

The aim of this study was to investigate the parallel mediating effect of CERS on the relationship between adolescents’ neuroticism and depressive symptoms. Although prior research has investigated the relationship among neuroticism, cognitive emotion regulation, and depressive symptoms, most of these studies have focused on one kind of CERS (e.g., rumination) in testing the relationship between neuroticism and depressive symptoms (10–12). Previous studies have not examined the roles of adaptive and maladaptive CERS simultaneously. Moreover, maladaptive and adaptive CERS were differentiated to test the protective and risk roles regarding the relationship between neuroticism and depressive symptoms. This approach could enable the development of good, effective intervention programs. To cope with stressful events, adolescents can use various CERS that can effectively prevent the development of internalizing symptoms. Therefore, compared with interventions for a stable personality trait (e.g., neuroticism), training adolescents to adopt adaptive CERS is more effective in reducing depressive symptoms. That is, the incidence of depressive symptoms can be decreased by providing training in adaptive, rather than maladaptive CERS.

In conclusion, the hypothesis of this study is that CERS would mediate the relationship between adolescent neuroticism and depressive symptoms. However, whether this mediation effect is partial or full requires further investigating. This study used adolescents as the research subjects, and structural equation modeling (SEM) was used to test the mediating roles of CERS in the relationship between neuroticism and depressive symptoms to better understand the psychological mechanisms of this relationship.

## Materials and Methods

### Subjects

The study was approved by the Academic Ethics Committee of Hunan University of Science and Technology. The parents agreed for their children to participate in the study by signing a consent form.

Classes were randomly selected from four middle schools in Anshan and Shaoyang in China, and the entire group was tested in class units. During formal testing, the researcher coordinated with the teachers to have the middle school students complete the questionnaire in the classroom and then collected them upon completion. From a total of 1,498 questionnaires distributed, 1,377 were retrieved. After the exclusion of invalid questionnaires based on the inclusion and exclusion criteria, 112 invalid questionnaires were eliminated (75 questionnaires with the same or regular responses to all of the questions and 37 questionnaires with at least half the data), yielding a final total of 1,265 valid questionnaires. The effective response rate for the questionnaire was 91.86%. A total of 648 boys accounted for 51.2% of the sample, whereas 617 girls accounted for 48.8%. The average age was 14.87 years old (*SD* = 1.24). A total of 223 students were in grade seven, accounting for 17.6%; 202 were eighth-grade students, accounting for 16%; 221 were ninth-grade students, accounting for 17.5%; 199 were high school freshmen, accounting for 15.7%; 211 were high school second-year students, accounting for 16.7%; and 209 senior high school students were in their third year, accounting for 16.5%.

### Procedure

First, a project training meeting was held to train the teachers and lab assistants depending on the unique mission, including using Mandarin to introduce the purpose and value of the research, distributing and collecting the questionnaires, and answering students’ questions. Second, all 1,498 students completed the CERQ-C, CDI-C, and EPQ-Ck consecutively. A final questionnaire included basic demographic characteristics, such as age, gender, and grade.

### Measures

#### CERQ-C

The CERQ-C revised by Wang et al. (15) was used. The questionnaire contains nine factors, namely rumination, self-blame, catastrophizing, other-blame, positive refocusing, positive reappraisal, refocus on planning, acceptance, and putting into perspective (15). Each subscale contains four questions for a total of 36 questions. The questionnaire uses a five-point Likert scale ranging from 1 (never) to 5 (always). Higher scores on a particular subscale are an indication of a greater frequency of using that cognitive emotion adjustment strategy. In this study, the Cronbach’s α values of the nine subscales were 0.69–0.77.

#### CDI-C

The CDI-C revised by Wu et al. (16) was used. The scale contains 27 questions and five factors: negative mood, anhedonia, ineffectiveness, negative self-esteem, and interpersonal problems (16). Each item contains three options that describe the degree of depression and are recorded as 0, 1, or 2 points. The higher that score on the questionnaire is, the more depressed that individual is. The questionnaire has been used to measure depressive symptoms in children and adolescents aged 7 to 17 years old. According to the relevant requirements of the Ethics Code for Psychology Professionals, the questionnaire used in this study excluded an item related to suicidal intentions. In this study, the Cronbach’s α of the CDI-C was 0.80.

#### EPQ-Ck

The neuroticism scale of the Chinese children’s edition of the Eysenck Personality Questionnaire revised by Gong was used (17). The subscale contains 23 questions using a two-point scoring method (1 for “yes”; 0 for “no”). The higher that score is, the higher that individual’s level of neuroticism is. The questionnaire is applicable for evaluating neurotic traits in children and adolescents aged 7 to 15 years old. The Cronbach’s α of the neuroticism scale in this study was 0.84.

### Statistical Analyses

**Figure 1:**
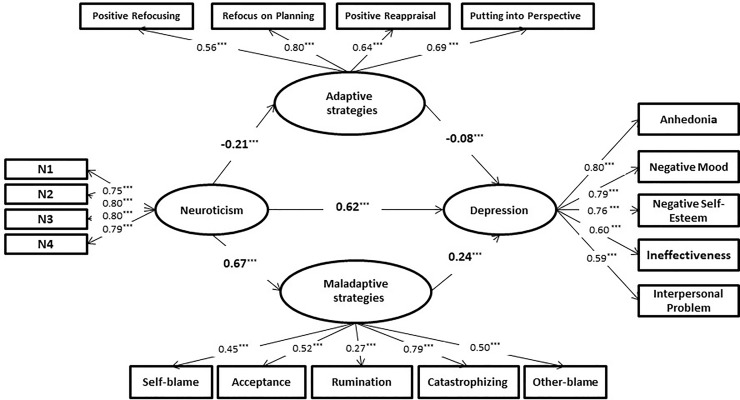
Standardized path coefficients of the mediation model. ^***^p < 0.001.

Partial correlation analysis was performed using SPSS software, version 24.0. SEM and parallel mediation analysis were conducted with Mplus software, version 8.0. Multigroup confirmatory factor analysis (CFA) was conducted with Amos software, version 24.0, to analyze structural invariance across gender and age groups. The model was composed of four latent variables: neuroticism, maladaptive CERS, adaptive CERS, and depressive symptoms (see **Figure 1**). This study used a MLR estimator to manage nonnormally distributed data. We used several goodness-of-fit indices to evaluate the SEM (18). A series of goodness-of-fit indices were selected for cross-group comparisons within the framework of CFA because equivalence of measures incurred nested models. Based on samples larger than 300, ΔCFI (< 0.01) and ΔRMSEA (< 0.015) were used to identify a significant decrease in fit between a series of progressively restricted models, as suggested by Chen (19).

## Results

### Correlation Analysis

Controlling for age and gender, we performed a partial correlation analysis of adolescent neuroticism, CERS, and depressive symptoms (see **Table 1**). The results affirmed a significant positive correlation among neuroticism, depressive symptoms, and acceptance (*ps* < 0.001), inconsistent with the results for studies of Western cultures (20). Certain emotions in the Eastern cultural context might be different from those in Western, English-speaking countries, rendering the avoidance of item bias difficult. Therefore, acceptance as a strategy should be regarded as a maladaptive cognitive emotion regulation strategy in this study. In addition, adaptive CERS (including positive refocusing, refocus on planning, positive reappraisal, and putting into perspective) and maladaptive CERS (including self-blame, acceptance, catastrophizing, rumination, and blaming others) had significant correlations with neuroticism and depressive symptoms (*ps* < 0.001). A significant, positive correlation among neuroticism, maladaptive CERS, and depressive symptoms was observed (*ps* < 0.01), whereas adaptive CERS had a significant, negative correlation with neuroticism and depressive symptoms (*ps* < 0.01).

**Table 1:** Partial correlations among the study variables while controlling for age and gender (*N* = 1,265).

Scale	Depressive symptoms	1	2	3	4	5	6	7	8	9	10	11
1	0.688^**^											
2	−0.214^**^	−0.196^**^										
3	0.471^**^	0.485^**^	0.048									
4	0.318^**^	0.260^**^	0.070^**^	0.624^**^								
5	0.308^**^	0.300^**^	0.054	0.705^**^	0.359^**^							
6	0.110^**^	0.143^**^	0.336^**^	0.555^**^	0.253^**^	0.265^**^						
7	−0.182^**^	−0.148^**^	0.735^**^	0.007	−0.037	0.023	0.219^**^					
8	−0.136^**^	−0.111^**^	0.816^**^	0.113^**^	0.119^**^	0.084^**^	0.316^**^	0.411^**^				
9	−0.232^**^	−0.231^**^	0.767^**^	−0.018	0.066^*^	0.021	0.298^**^	0.455^**^	0.498^**^			
10	−0.100^**^	−0.107^**^	0.750^**^	0.047	0.071^*^	0.038	0.193^**^	0.365^**^	0.593^**^	0.386^**^		
11	0.487^**^	0.506^**^	−0.153^**^	0.744^**^	0.334^**^	0.365^**^	0.187^**^	−0.121^**^	−0.048	−0.224^**^	−0.071^*^	
12	0.273^**^	0.332^**^	−0.153^**^	0.563^**^	0.032^**^	0.250^**^	0.052	−0.062^*^	−0.111^**^	−0.214^**^	−0.080^**^	0.450^**^

*p < 0.05, ^**^p < 0.01, 1, Neuroticism; 2, Adaptive CERS; 3, Maladaptive CERS; 4, Self-blame; 5, Acceptance; 6, Rumination; 7, Positive refocusing; 8, Refocus on planning; 9, Positive reappraisal; 10, Putting into perspective; 11, Catastrophizing; 12, Other-blame.

### Testing the Mediating Effect

To control for instability in the scale and to reduce the increased measurement error caused by multiple items of the potential variables on the neuroticism subscale, this study used item parceling for observable variables of neuroticism in SEM (21). Initially, unidimensional CFA was performed on the neuroticism subscale. According to the item-to-construct balance method (22), four item parcels (namely N1, N2, N3, and N4) were created (see **Figure 1**) and the 23 items were arranged on the basis of factor loadings from high to low. Factor loading represents how well a particular item measures the factor on which it loads. Based on the equalization of the item factor loadings of each parcel, the items were ranked from high to low into the first item parcel, into the second item parcel, and so on. The first group contained items 1 (1 refers to the item with the highest factor loading), 8, 9, 16, and 17. The second group contained items 2, 7, 10, 15, 18, and 23. The third group contained items 3, 6, 11, 14, 19, and 22. The fourth group contained items 4, 5, 12, 13, 20, and 21. Then, the average score of the items included in each parcel was calculated. In addition, the total scores for four of the adaptive CERS were calculated separately, as an observational indicator of the adaptive emotion regulation strategy. The total scores of the five maladaptive CERS were calculated separately as an observational indicator of the maladaptive emotional regulation strategy. The total scores were calculated from the five subscales of CDI-C as the observational indicators of depressive symptoms.

Building the model of the direct effect of adolescent neuroticism on depressive symptoms (direct-effect model) and the mediating role of CERS in the link between adolescent neuroticism and depressive symptoms (mediation model) separately, we used goodness-of-fit indices to evaluate the SEM (see **Table 2** and **Figure 1**). **Table 2** shows an excellent fit to the data. The TLI and CFI are greater than 0.90 in both models, and the RMSEA and SRMR are less than 0.80. From the goodness-of-fit indices of the direct-effect model, adolescent neuroticism positively predicted depressive symptoms *β* = 0.80 (*p* < 0.001). From the mediation model, adolescent neuroticism had a significantly positive predictive effect on maladaptive CERS *β* = 0.62 (*p* < 0.001). Adolescent neuroticism also had a significantly negative predictive effect on adaptive CERS *β* = −0.21 (*p* < 0.001). Moreover, maladaptive CERS had a significantly positive predictive effect on adolescent depressive symptoms *β* = 0.24 (*p* < 0.001), and adaptive CERS had a significantly negative predictive effect on adolescent depressive symptoms *β* = −0.08 (*p* < 0.01). Adolescent neuroticism had a significantly positive predictive effect on depressive symptoms *β* = 0.62 (*p* < 0.001). After the two types of CERS were introduced into the model, the direct predictive effect of adolescent neuroticism on depressive symptoms decreased from 0.80 to 0.62, but it remained still significantly different from zero (*p* < 0.001). According to the evaluation criteria for intermediary analysis, these findings indicated that CERS played a partial parallel mediating roles in the relationship between adolescent neuroticism and depressive symptoms.

**Table 2:** Fit indices of the two models.

Model	*χ^2^/df*	TLI	CFI	RMSEA	SRMR
Direct-effect model	3.80^***^	0.982	0.987	0.047	0.022
Mediation model	6.66^***^	0.901	0.915	0.067	0.064

***P < 0.001; TLI, non-normed fit index; CFI, comparative fit index; RMSEA, root mean square error of approximation; SRMR, standardized root mean square residual.

This work used the nonparametric percentile bootstrap method using bias correction to examine the indirect effects of the latent variable model, and 95% confidence intervals were obtained. The bootstrap confidence intervals for maladaptive CERS and adaptive CERS were (0.209, 0.490) and (0.010, 0.065), respectively. The confidence intervals did not include 0, indicating that the indirect effects were significant. The effect size was based on the ratio of the mediating effect to the total effect. The effect size was 20.3% for maladaptive CERS and 2.1% for the adaptive CERS.

### Structural Invariance Testing

Given that the use of CERS differed by gender and age, subsequent analyses used multigroup CFA to examine the gender- and age-related structural invariance in the CERS in their parallel mediating roles between neuroticism and depressive symptoms. In the present study, the respondents were divided into two age groups: junior high school students (*n* = 646) and high school students (*n* = 619). Since the test of structural invariance involves comparing the difference between the baseline model and the nested model for evaluating the invariance fit, in addition to the commonly used fitting indices, such as *χ^2^*, GFI, CFI, and RMSEA, incremental fit indices reflecting the differences between models were also needed. Given that Δ*χ^2^* is sensitive to sample size, even if the model and the observation matrix fit well, Δ*χ^2^* is easily statistically significant in the case of large samples, thereby rejecting the correct theoretical model. In contrast, ΔCFI and ΔRMSEA are not affected by the sample size. When ΔCFI < 0.01 and ΔRMSEA < 0.015, the model groups are equivalent (17). Differences in multiple sets of comparisons were primarily assessed by ΔCFI and ΔRMSEA because of the large sample size. The results showed that the comparison of the nested models (see **Table 3**), factor loadings, factor variance–covariance, and mediation model of the measurement residuals were basically the same, indicating consistency across genders and ages.

**Table 3:** Tests of structural invariance.

Types	Model	*χ^2^*	*df*	GFI	CFI	RMSEA	SRMR	*ΔCFI*	*ΔRMSEA*
Gender									
Mediation effect model	Model 1	1002.892^***^	260	0.914	0.915	0.048	0.072		
	Model 2	1017.210^***^	274	0.913	0.915	0.046	0.073	0.000	0.002
	Model 3	1024.644^***^	279	0.912	0.915	0.046	0.073	0.000	0.000
	Model 4	1024.951^***^	280	0.912	0.915	0.046	0.076	0.000	0.000
Age									
Mediation effect model	Model 1	1034.429^***^	260	0.913	0.911	0.049	0.065		
	Model 2	1045.125^***^	274	0.912	0.911	0.047	0.065	0.000	0.002
	Model 3	1054.435^***^	279	0.911	0.911	0.047	0.068	0.000	0.000
	Model 4	1067.364^***^	280	0.910	0.909	0.047	0.068	0.002	0.000

^***^p < 0.001; Model 1: baseline model, free estimation of each parameter; Model 2: the constraint factor load was equal between groups; Model 3: the constraint factor variance–covariance was equal between groups; Model 4: the limit of measurement residuals was equal between groups.

## Discussion

### Correlation Among Neuroticism, CERS, and Depressive Symptoms

Partial correlation analysis of the links among adolescent neuroticism, CERS, and depressive symptoms affirmed a significant, positive correlation among neuroticism, maladaptive CERS, and depressive symptoms, as well as various dimensions of each of the factors. A significant negative correlation was also observed of adaptive CERS with neuroticism and depressive symptoms, as well as with each dimension.

Neuroticism is a relatively stable personality trait that includes the negative emotional experiences associated with the tendency of these individuals to avoid stress, to engage in catastrophic thinking regarding stressful events, and to have emotional disorders. Given the weak ability to regulate negative emotions, individuals with high levels of neuroticism commonly experience negative emotions, such as anger, nervousness, depressive symptoms, and anxiety, which are characterized by impulsivity, ease of emoting, and anxiety (23). Studies have shown that neuroticism is an important risk factor across certain emotional disorders and personality disorders. Neuroticism is also an independent predictor of pathological psychology, such as depressive symptoms and anxiety (24). Compared to individuals with low levels of neuroticism, those with high levels of neuroticism are more prone to being depressed because they experience more stressful events. In real life, teenagers with high levels of neuroticism are likely to have psychological problems, such as loneliness and social anxiety. These teenagers also have low perceptions of social support, for example, from family and peers. In the process of interacting with teachers and peers, they often experience a sense of frustration, leading to bad relationships. Therefore, a significant, positive correlation exists between adolescent neuroticism and depressive symptoms.

Partial correlation analysis also found that neuroticism was closely related to specific CERS. In a past study, Li et al. showed that levels of neuroticism could affect the use of different CERS among adult subjects (25). For instance, people with high levels of neuroticism generally use rumination strategies in attempts at negative emotion regulation caused by stressful events. Some scholars have even regarded meditation as a cognitive manifestation of neuroticism (26). Studies have also described a mediating role of rumination in the relationship between neuroticism and depressive symptoms (10). Hasking et al. found a higher positive correlation between neuroticism and cognitive reassessment in adolescents compared with that in adults (27). In addition, some studies have used cognitive neuroimaging techniques to explore the brain mechanisms of the relationship between neuroticism and cognitive regulation strategies. For instance, Canli et al. (28) found a positive correlation between neuroticism and the degree of activation of the dorsolateral prefrontal cortex (dlPFC) during a task in which subjects viewed a set of emotional pictures. In addition, a negative correlation exists between levels of neuroticism and the degree of cingulated gyrus (ACC) activation in “oddball detection” tasks (28). The dorsolateral prefrontal cortex and cingulate gyrus are considered important brain regions for emotional and cognitive functions, and they are the brain regions responsible for emotion regulation. The activation of the dorsolateral prefrontal cortex was enhanced when individuals with high levels of neuroticism used cognitive strategies to regulate emotion. However, high levels of neuroticism were not associated with activation of the ventrolateral prefrontal cortex (vlPFC) during cognitive emotion regulation tasks, despite being an important brain region for effective cognitive emotion regulation. Therefore, adolescents with high levels of neuroticism might be more inclined to adopt maladaptive CERS to regulate emotion. The findings of this study are consistent with the previous literature, demonstrating that adolescent neuroticism is closely related to cognitive emotion regulation.

Studies have shown that individuals with depressive symptoms have difficulty using effective emotional regulation strategies. Compared with normal individuals, depressed individuals use fewer adaptive emotional regulation strategies to address negative moods (29). In addition, cognitive neuroscience studies have found that depressive symptoms are associated with abnormal patterns of activity in the brain regions responsible for adaptive emotion regulation (30). Garnefski et al. (23) confirmed that positive refocusing and refocusing on plan is negatively associated with depressive symptoms in children aged 9 to 11 years old (24), whereas self-blame, catastrophizing, and other blame are positively associated with depressive symptoms in children aged 9 to 11 years old (20). The Chinese scholar Luo Fusheng et al. also affirmed a close relationship between CERS and depressive symptoms in junior and senior high school and college students (7). Adolescence is a period during which depressive symptoms are frequently experienced, and these symptoms are a precursor to depression. The excessive use of maladaptive emotional regulation strategies can increase adolescents’ negative emotional experiences and the manifestation of depressive symptoms. In addition, although the strategy of acceptance is considered an adaptive cognitive emotion regulation strategy in Western cultures, there was a positive correlation between depression symptoms and this strategy in a sample of college students from China (7). The same results were obtained in this study, most likely due to item bias.

### Mediating Roles of CERS

Previous studies have mainly examined the role of a type of CERS in the relationship between neuroticism and depressive symptoms in adolescents and adults. For instance, rumination plays a mediating role in the link between neuroticism and depressive symptoms (11, 12). However, there has been no further exploration of the early occurrence and mode of development of this psychological mechanism. This study used SEM to explore the mechanisms of adolescent neuroticism’s effects on depressive symptoms, and it employed multiple mediation models to examine the mediation effects of two types of CERS. Moreover, group CFA was used to examine the age and gender invariance of the mediating effects. The results indicated that cognitive emotion regulation partially mediated the relationship between adolescent neuroticism and depressive symptoms, showing the following. (1) Neuroticism had an indirect effect on depressive symptoms through mediation by CERS; that is, adolescents with high levels of neuroticism adopted fewer adaptive or more maladaptive CERS and then developed depressive symptoms. (2) Neuroticism had a strong, direct, predictive effect on depressive symptoms, and the indirect effects (20.3%) produced by maladaptive CERS were greater than the indirect effects (2.1%) produced by adaptive CERS. In addition, regarding the path coefficients, neuroticism had a stronger influence on maladaptive CERS than adaptive CERS. The effect of maladaptive CERS on depressive symptoms was also greater than that of adaptive CERS. The group CFA results showed that the parallel mediating effects of the two types of CERS were invariant across age and gender, indicating that mediation is similar with respect to developmental stages and genders.

For adolescents with high levels of neuroticism, the overuse of maladaptive CERS can increase susceptibility to depressive symptoms by exacerbating negative emotional states, thereby leading to the development of depressive symptoms. Ling et al. found that rumination mediated the effect of neuroticism on depressive symptoms in high school students (11). Furthermore, in the Western cultural context, maladaptive emotional regulation strategies, including rumination, inhibition of thought, and inhibition of expression, fully mediated the effect of neuroticism on depressive symptoms (31). Given that this work examined only CERS and did not include behavioral types of emotional regulation strategies, the use of CERS played a partially mediating role only in the early stage of the development of neuroticism on depressive symptoms. Future research could further examine whether maladaptive emotional regulation strategies, such as expression suppression strategies at the behavioral level (32), play a mediating role in the link between neuroticism and depressive symptoms in Eastern cultural contexts.

In this study, the indirect effect of adaptive CERS on the relationship between neuroticism and depressive symptoms was 2.3% of the total effect, accounting for less than 5% of the total effect, indicating that the use of adaptive CERS had a limited effect on adolescent depressive symptoms (33). Therefore, reducing the use of maladaptive CERS and increasing the use of adaptive CERS are important for relieving depressive symptoms in adolescents. In addition, this study used cross-sectional methods, and the results are thus susceptible to individual response bias and common method bias. Therefore, future research could use multitrait-multimethod analysis to verify the results of this study and investigate the causal relationships between the variables through a longitudinal study. Furthermore, without the participation of psychiatrists, the diagnosis of neuroticism, anxiety or depression could not be confirmed, hampering deeper exploration of the results. Therefore, the accuracy of psychiatric diagnoses made by psychiatrists should be applied to improve the explanatory power of our model in future studies. Moreover, given that some of the subjects in this study were older than the suitable population for the EPQ-CK, other studies could further expand the sample range to include the EPQ-C to repeat the results of this study. Finally, because of the difficulty in changing relatively stable personality traits, subsequent research could design interventions from the perspective of CERS, thereby reducing cases of depressive symptoms in adolescents and improving their mental health.

## Data Availability Statement

The datasets generated for this study are available on request to the corresponding author.

## Ethics Statement

The studies involving human participants were reviewed and approved by the institutional review board of Hunan University of Science and Technology. Written informed consent to participate in this study was provided by the participants’ legal guardian/next of kin.

## Author Contributions

CL and LC reviewed the literature and wrote the paper. SC outlined the structure of the paper, reviewed the literature, and wrote the paper.

## Funding

This research was supported by National Education Scientific Planning Projects of China (DBA180316) and the Scientific Research Fund of the Hunan Provincial Education Department (16K034), awarded to CL; and the Liaoning Planning Office of Philosophy and Social Science Planning Foundation (L18WSZ011) and University of Science and Technology Liaoning Talent Project Grants (601011507-33) awarded to LC.

## Conflict of Interest

The authors declare that the research was conducted in the absence of any commercial or financial relationships that could be construed as a potential conflict of interest.
